# Fluid intake patterns: an epidemiological study among children and adolescents in Brazil

**DOI:** 10.1186/1471-2458-12-1005

**Published:** 2012-11-20

**Authors:** Rubens Feferbaum, Luiz Carlos de Abreu, Claudio Leone

**Affiliations:** 1 Departamento de Pediatria, Faculdade de Medicina da Universidade de São Paulo. FMUSP, São Paulo, SP, Brazil; 2 Departamento de Saúde Materno-Infantil, Faculdade de Saúde Pública da USP, São Paulo, SP, Brazil; 3 Laboratório de Delineamento de Estudos e Escrita Científica, Faculdade de Medicina do ABC, Santo André, São Paulo, Brazil

**Keywords:** Infant nutrition, Childhood obesity, Carbohydrate consumption, Nutritional education, Fluid intake, Liquid Kcal, Water intake

## Abstract

**Background:**

Energy from liquids is one of the most important factors that could impact on the high prevalence of children and adolescents obesity around the world. There are few data on the liquid consumption in Brazil. The aim of this study is to evaluate the volume and quality of liquids consumed by Brazilian children and adolescents and to determine the proportion of their daily energy intake composed of liquids.

**Methods:**

A multicenter study was conducted in five Brazilian cities; the study included 831 participants between 3 and 17 years of age. A four-day dietary record specific to fluids was completed for each individual, and the volume of and Kcal from liquid intake were evaluated. The average number of Kcal in each beverage was determined based on label information, and the daily energy intake data from liquids were compared with the recommendations of the National Health Surveillance Agency (Agência Nacional de Vigilância Sanitária– ANVISA), the Brazilian food regulation authority, according to each subject’s age.

**Results:**

As the children aged, the volume of carbonated beverages that they consumed increased significantly, and their milk intake decreased significantly. For children between the ages of 3 and 10, milk and dairy products contributed the greatest daily number of Kcal from liquids. Sugar sweetened beverages which included carbonated beverages, nectars and artificial beverages, accounted for 37% and 45% of the total Kcal from liquid intake in the 3- to 6-year-old and 7- to 10- year-old groups, respectively. Among adolescents (participants 11- to 17- years old), most of the energy intake from liquids came from carbonated beverages, which accounted for an average of 207 kcal/day in this group (42% of their total energy intake from liquids). Health professionals should be attentive to the excessive consumption of sugar sweetened beverages in children and adolescents. The movement toward healthier dietary patterns at the individual and population levels may help to improve programs for preventing overweight and obesity in children and adolescents.

**Conclusion:**

From childhood to adolescence the daily volume of liquid ingested increased reaching a total of 2.0 liters on average. Of this volume, the daily volume of milk ingested decreased while the carbonated drinks, sweetened, nectars and artificial beverages increased significantly. The proportion of water remained constant in about 1/3 of the total volume. From 3 to 17 years of age the energy intake from carbonated beverages increased by about 20%. The carbonated drinks on average corresponded to a tenth of the daily requirements of energy of adolescents.

## Background

Obesity is considered an important public health problem in developed countries and a global epidemic, according to World Health Organization (WHO) [1]. Current data shows that the prevalence of overweight among children and adolescents is high also in developing countries [1-4].

The prevalence of obesity in children and adolescents, their biopsychosocial impact, high rates of failure in the treatment of obesity in adulthood and greater risk of obese child becoming an obese adult, highlights obesity as one of the major nutritional problems in childhood [5].

In Brazil, the 2008-2009 Consumer Expenditure Survey (*Pesquisa de Orçamentos Familiares*), a government study that analyzes household spending and consumption according to income, indicates that the proportion of 10- to 19-year-old boys who are overweight increased from 3.7% in 1974-75 to 21.7% in 2008-09; for girls in the same age group, the proportion increased from 7.6% to 19.4% during the same time period [6].

According to Monteiro et al [7]., the number of overweight children has tripled in Brazil in the last 30 years, while in the United States it has doubled. This increase was most important in Southern and Southeastern Brazil, on the most advantaged population. Therefore, excess weight is already an important nutritional shift by its intensity and frequency.

Although obesity is a multifactorial disorder, one of the strategies that issued to reduce obesity involves decreasing the energy intake from added sugars in an individual’s diet [8]. Studies have shown that a significant increase in energy intake among children and adolescents arises from the prevalent consumption of sugars added to processed foods and beverages [9-13].

Regular consumption of sugar calories in liquid form is said to be responsible for body weight gain due to their low satiety and high added sugar content [8] Studies have shown high consumption of sugar-sweetened beverages in children and adolescents [12-19], which appears to be a critical component among the potential environmental and social factors implicated in the obesity epidemic [20], beyond the effects of these added sugars on cardiovascular risk and type 2 diabetes mellitus in children and adults [14-18].

Epidemiological studies are generally designed to evaluate solid food intake [19,21], whereas, beverages should also be considered in the nutritional approach of individuals and populations, specially on tropical environments as Brazil, where high temperatures can contribute to dehydration.

For this reason, the purpose of this study was to investigate the volume and quality of the beverages consumed by individuals within the pediatric age range (from 3 to 17 years of age).

## Methods

This descriptive, study included 831 children and adolescents of both genders who ranged from 3 and 17 years of age and resided in five Brazilian cities: São Paulo, Belo Horizonte, Porto Alegre, Rio de Janeiro and Recife.

The universe sampled for this survey represents the Brazilian children and adolescents resident in the urban areas. The sample was calculated based on the population proportions per each age groups (3-6 years old, 7-10 years old and 11-17 years old) and gender.

Census tracts are territorial units defined by IBGE [22] (Brazilian Institute of Geography and Statistics) to guide the population spatial distribution, following collection data criteria, in which only one census surveyor can cover the whole area.

From the universe of census tracts in the major Brazil urban areas, 90 census tracts were randomly selected reaching the calculated sample size, in order to represent the socioeconomic diversity, according the Human Development Index (HDI): 1: tertile of low socioeconomic level, 2: tertile of medium socioeconomic level and 3: tertile of high socioeconomic level.

The methodology and purpose of the study were presented to the potential participants and those whom responsible consented to participate in the study, signed the term and were, therefore, included in the sample. Information about the study and questionnaires to complete, were given to the participants who agreed to participate. All incomplete questionnaires were excluded from the study.

The liquid intake of participants was assessed quantitatively and qualitatively using a dietary record that was given to each child’s caretaker during the first home visit and completed over a four-day period. The caregivers were instructed to maintain the children’s usual dietary habits and to record their daily liquid intake. At school, participants’ teachers helped to complete the questionnaires.

When a liquid was ingested, the time, type and volume of the beverage were recorded using photographs of the utensils and containers used, which increased data reliability. The liquids were classified into 11 groups: water, flavored water, milk and dairy products, hot beverages (including coffee and tea), carbonated beverages (soft drinks), natural juices (fruit juices with no added sugar), artificial beverages (containing food coloring, flavoring and added sugar), nectars (fruit pulp with added sugar), functional beverages (energy and isotonic drinks), alcohol and others, which included soy-based beverages and instant soups, for example.

To assess the daily energy contribution of each type of beverage, the average number of Kcal was determined based on the labels of the three most-consumed brands in the market, according to a study by Kantar WorldPanel (2010) [20].

Beverages for which no nutritional information label was available, the number of Kcal was calculated using the food composition table of the State University of Campinas (*Universidade Estadual de Campinas*) [23]. These beverages included fruit smoothies, fresh-squeezed orange juice, fresh coconut water and sugarcane juice.

The sugar-sweetened beverages (SSB) group included carbonated beverages, artificial beverages and nectars, which contain a high concentration of added sugar. Although natural fruit juices contain sugars, they do not fall into the SSB category because they contain sugars from the fruit itself in addition to nutritional and functional elements.

The energy intake from liquids of each participant was compared with the daily energy requirements recommended for children and adolescents by the Brazilian food regulation authority, the National Health Surveillance Agency (Agência Nacional de Vigilância Sanitária – ANVISA). These comparisons were made for each age group [24].

The study protocol was approved by the Research Ethics Committee at the Darcy Vargas Children’s Hospital.

### Statistical analysis

The means, standard deviations and minimum and maximum values for the volume and energy content of each beverage were calculated.

An ANOVA procedure and a student’s t-test were used to compare the mean volume and energy intake for the different genders and age groups.

## Results

The characteristics of the study population, according to gender, age group and geographical location, are shown in Table 1.

**Table 1:** Sample characteristics according to gender, age group and city of residence

	**n**	**%**
**Gender**		
Boys	422	51
Girls	409	49
**Age - Boys**		
3 to 6 years	130	31
7 to 10 years	97	23
11 to 17 years	195	46
**Age - Girls**		
3 to 6 years	127	31
7 to 10 years	93	23
11 to 17 years	189	46
**Cities**		
Belo Horizonte	156	19
Porto Alegre	161	20
Recife	171	20
Rio de Janeiro	181	21
São Paulo	162	20

**Figure 1:**
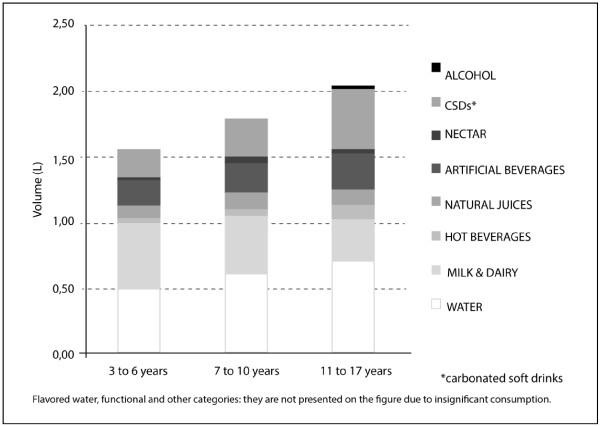
Mean volume intake for each category of fluids according to age.

Gender did not significantly affect the volume of liquid intake. It was found that the total volume of liquid intake increased with age, as did the volume of water and carbonated beverages consumed (p<0.05), whereas the intake of milk and dairy products decreased as the age of the participants increased (p<0.05; Figure 1 and Table 2).

**Table 2:** Intake volume (L) for each beverage category according to age

	**3 to 6 years**	**7 to 10 years**	**11 to 17 years**	**p value**
**TOTAL**	1.57 ± 0.63 (0.45-4)	1.81 ± 0.64 (0.5-3.79)	2.05 ± 0.92 (0.4-5.78)	0.001
**WATER**	0.48 ± 0.37 (0-2.50)	0.61 ± 0.36 (0-2.31)	0.70 ± 0.56 (0-3.90)	0.001
**MILK & DAIRY**	0.51 ± 0.33 (0-2.71)	0.43 ± 0.26 (0-1.35)	0.32 ± 0.27 (0-1.30)	0.001
**HOT BEVERAGES**	0.03 ± 0.09 (0-0.70)	0.05 ± 0.14 (0-1.10)	0.11 ± 0.2 (0-1.82)	0.001^1^
**FLAVORED WATERS**	0.01 ± 0.04 (0-0.50)	0.01 ± 0.07 (0-0.75)	0.01 ± 0.08 (0-1.50)	0.797
**NATURAL JUICES**	0.10 ± 0.16 (0-1.20)	0.14 ± 0.24 (0-1.20)	0.11 ± 0.22 (0-1.31)	0.206
**ARTIFICIAL BEVERAGES**	0.18 ± 0.26 (0-1.60)	0.22 ± 0.29 (0-1.50)	0.27 ± 0.36 (0-2.28)	0.001^2^
**NECTAR**	0.03 ± 0.09 (0-0.75)	0.04 ± 0.11 (0-0.70)	0.03 ± 0.1 (0-0.80)	0.461
CSDs*	0.21 ± 0.23 (0-1.40)	0.30 ± 0.34 (0-1.85)	0.48 ± 0.42 (0-2.91)	0.001
**FUNCTIONAL DRINKS**	0 ± 0.01 (0-0.24)	0	0.01 ± 0.05 (0-0.56)	0.102
**ALCOHOL**	0	0	0.01 ± 0.11 (0-1.75)	0.095
**OTHERS**	0 ± 0.03 (0-0.23)	0 ± 0.04 (0-0.33)	0 ± 0.01 (0-0.20)	0.022^3^

*CSDs: Carbonated soft drinks.

^1^ p<0.05for3- to 6-year-oldsand 11- to 17-year-olds and for 7- to 10 –year-olds and 11- to 17-year-olds.

^2^ p<0.05for 3- to 6-year-olds and 11- to 17-year-olds.

^3^ p<0.05 for 3- to 6-year-olds and 11- to 17-year-olds and for 7- to 10-year-olds and 11- to 17-year-olds.

For study participants aged 3 to 6 years, 7 to 10 years and 11 to 17 years, water intake represented 31%, 33% and 34% (p<0.05) of the total volume of liquid intake, respectively; carbonated beverages composed 13%, 17% and 23% of that figure (p<0.05), respectively; and milk and dairy products represented 32%, 24% and 16% of that figure (p<0.05), respectively.

Regarding energy intake, there were no significant differences between genders. However, energy intake increased with age. Milk and dairy products were the major contributors to energy intake from beverages for children aged 3 to 6 and 7 to 10 years old, with a decreasing trend associated with increases in age (p<0.05).

**Figure 2:**
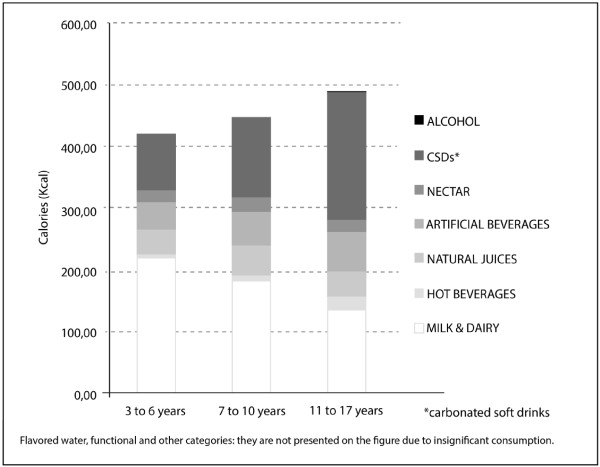
Mean energy intake according to the volume of each beverage category.

This beverage category is, naturally, the major source of energy in childhood as they provide important macro nutrients, essential to bone development. The next most significant contributor was the carbonated beverage category within that age group. However, in adolescents aged 11 to 17 years old, carbonated beverages were the major source of Kcal from liquids (on average, individuals in this age group consumed 207.08kcal/day from carbonated beverages, thus deriving 42% of their daily energy intake from beverages and 10% of their recommended daily energy intake). Artificial beverages constituted a significant source of Kcal in all age groups (Figure 2, Table 3 and 4).

**Table 3:** Energy intake (kcal) for each beverage category according to age

	**3 to 6 years**	**7 to 10 years**	**11 to 17 years**	**p**
**TOTAL**	421.6 ± 203. 57 (64-1565.60)	451.04 ± 229.02 (47.9-1376.20)	490.54 ± 262.64 (22.8-1845.48)	0.001^1^
**FLAVORED WATERS**	1.36 ± 9.29 (0-106)	1.88 ± 13.56 (0-159)	0.39 ± 3.18 (0-44.40)	0.112
**MILK & DAIRY**	218.71 ± 159.87 (0-1427.60)	181.53 ± 122.03 (0-749.60)	134.23 ± 122.31 (0-589.65)	0.001
**HOT BEVERAGES**	6.02 ± 16.62 (0-131.60)	8.98 ± 25.32 ± (0-206.80)	21.27 ± 39.18 (0-342.35)	0.001^2^
**NATURAL JUICES**	37.91 ± 61.72 (0-441.60)	49.97 ± 87.15 (0-441.60)	41.38 ± 83.39 (0-483)	0.262
**ARTIFICIAL BEVERAGES**	45.47 ± 63.58 (0-364.80)	51.56 ± 68.53 (0-342)	63.58 ± 84.09 (0-521.55)	0.008^3^
**NECTAR**	18.09 ± 52.91 (0-420)	23.87 ± 62.36 (0-392)	19.32 ± 59.28 (0-448)	0.556
CSDs*	92.49 ± 99.25 (0-593.60)	131.42 ± 146.17 (0-794.60)	207.08 ± 183.58 (0-1279.38)	0.001
**FUNCTIONAL DRINKS**	0.21 ± 3.36 (0-53.92)	0	1.51 ± 14.75 (0-237.30)	0.145
**ALCOHOL**	0	0	1.14 ± 14.17 (0-231)	0.234
**OTHERS**	1.32 ± 7.79 (0-61.20)	1.83 ± 10.27 (0-88.40)	0.25 ± 3.47 (0-54.40)	0.022^4^

*CSDs: Carbonated soft drinks.

^1,2,3,4^ p<0.05for 3- to 6-year-olds and 11- to 17-year-olds and for 7- to 10-year-olds and 11- to 17-year-olds.

**Table 4:** **Energy intake percentage provided by each beverage according to the recommended daily intake (RDI) values**[21]

		**TOTAL**	**FLAVORED WATERS**	**MILK & DAIRY**	**HOT BEVERAGES**	**NATURAL JUICES**	**ARTIFICIAL BEVERAGES**	**NECTAR**	CSDs*	**FUNCTIONAL DRINKS**	**ALCOHOL**	**OTHER**
3-year-olds	%	42.96	0	25.40	0.37	3.76	4.63	1.09	7.66	0	0	0.05
4- to 6-year-olds	%	28.56	0.12	14.24	0.45	2.59	3.08	1.36	6.59	0.02	0	0.11
7- to 10-year-olds	%	25.77	0.11	10.37	0.51	2.86	2.95	1.36	7.51	0	0	0.10
11- to 17-year-olds	%	24.53	0.02	6.71	1.06	2.07	3.18	0.97	10.35	0.08	0.06	0.01

*CSD: Carbonated soft drinks.

Sugar sweetened beverages (SSBs), including carbonated beverages, nectars and artificial beverages, provided 37%, 46% and 59% of the energy intake of individuals ages 3 to 6 years, 7 to 10 years and 11 to 17 years (p<0.05), respectively, whereas milk and dairy products contributed 52%, 40% and 27% (p<0.05) of the total energy intake for these age groups, respectively. Among 11- to 17-year-olds, SSBs alone contributed an average of 289.98 kcal/day, which constituted 14.5% of the recommended daily energy for this age group (Figure 2 and Table 4).

## Discussion

The results of this study indicate that a high daily energy intake results from high energy-density SSBs in school-age children and adolescents (Figure 2). When such patterns are associated with a high-energy solid food diet, they may lead to excessive weight gain.

Carbonated beverages yielded the greatest energy intake among adolescents: on average, 207.08 kcal/day, which is equivalent to 10% of the recommended daily energy intake for individuals within that age group (Table 4). Similar results were found in a study in the United States, in which soft drinks were found to be the main source of Kcal for 14- to 18-year-olds [19]. In the present study, the early introduction of SSBs into the diet is exacerbated by age and contributes with 156.05 kcal, 206.85 kcal and 289.98 kcal on average within the 3- to 6-year-old, 7- to 10-year-old and 11- to 17-year-old groups, respectively.

These results are similar to those obtained by Wang et al [25]. The energy contribution on 7- to 10-year-old and 11- to 17-year-old groups correspond to 12% and 15%, respectively, based on the recommended daily intake of energy; both percentages exceed the World Health Organization energy recommendation for free sugars in the total diet (10%), including solid foods [26].

Regarding the impact of tropical environment on fluid consumption, Brazil is located mostly in the intertropical zone (between the Equator, which passes through Macapa, and the Tropic of Capricorn, which passes through São Paulo), the hottest in the Earth. With a predominance of low altitudes, there are in Brazil hot climate varieties, with averages above 20 Celsius degrees [27].

Thus, children and adolescents resident in tropical areas should drink more water to replenish fluid losses caused by high temperatures and excessive sweating, in all regions of Brazil. Moreover, increase in water consumption should be encouraged in pediatric population due to their less tolerance to heat than adults, resulting in rapid increase in temperature, by virtue of the slower capacity of acclimatization [28].

However, the hydration should be through water consumption, which has no additives or calories. The present study found that participants opted for other beverages besides water, possibly increasing the amount of energy consumed (Table 2 and 3).

Popkin et al [29]. indicate that energy intake is significantly lower in individuals who regularly drink water, which helps to reduce obesity. A study of children in Germany found that over a one-year period, an increase in water consumption was associated with a 31% reduction in the risk of obesity [30]. Furthermore, water consumption before and during meals decreases feelings of hunger and increases satiety [29-31].

One important finding is that with age, carbonated beverage consumption increases and milk and dairy product intake decreases (Figure 1 and Table 2). Such findings have also been reported in other countries [9,32].

It is worrisome when the substitution of dairy products for carbonated beverages begins early in childhood because calcium intake positively affects bone mass and is essential for adequate bone development in childhood and adolescence [33-36].

Thus, the obesity epidemic in children and adolescents is a current public health challenge. Reducing energy intake may prove to be a determining factor in decreasing the prevalence of obesity.

Obesity is a major contributor to the rise of both illnesses and chronic disabilities. The world is facing illness characteristics of the modern era, including obesity, osteoporosis, cancer and diabetes [37]. Often coexisting in developing countries with malnutrition, obesity is a complex condition with serious social and psychological dimensions, affecting virtually all ages and socioeconomic groups [38]. Also low income preschool children are in an advanced stage of nutritional transition with a high prevalence of overweight [39].

The development of new eating habits as well as current trends in production and consumption, impacts on health, environment and social. With an overall increase in the prevalence of obesity, nutrition should take a leading role in both the prevention and treatment of these diseases.

The findings of this study reflect the issues with the quality and volume of beverages (especially SSBs) consumed by children and adolescents. Our findings should encourage healthcare professionals to be more diligent in recording liquid intake, as the latter may result in excess sugar consumption, especially when it is associated with a poor solid food diet. Natural juice comsumption and water intake to proper hydration must be encouraged to ensure healthy beverage. Individual guidance and educational programs centered on healthy eating are an important means of preventing childhood obesity and its complications in adulthood.

## Conclusion

From childhood to adolescence the daily volume of liquid ingested increased reaching a total of 2.0 liters on average. Of this volume, the daily volume of milk ingested decreased while the carbonated drinks, sweetened, nectars and artificial beverages increased significantly. The proportion of water remained constant in about 1/3 of the total volume. From 3 to 17 years of age the energy intake from carbonated beverages increased by about 20%. The carbonated drinks on average corresponded to a tenth of the daily requirements of energy of adolescents.

## Abbreviations

SSB: Sugar-sweetened beverages; CSD: Carbonated soft drinks; ANVISA: National Health Surveillance Agency [*Agência Nacional de Vigilância Sanitária*]; RDI: Recommended daily intake.

## Competing interests

The authors declare that they have no competing interests.

## Authors’ contributions

All authors participated in the acquisition of data and revision of the manuscript. RF, LCA and CL conceived of the study, determined the design, performed the statistical analysis, interpreted the data and drafted the manuscript. All authors read and gave final approval for the version submitted for publication.

## Pre-publication history

The pre-publication history for this paper can be accessed here:

http://www.biomedcentral.com/1471-2458/12/1005/prepub
